# Pediatric Sarcoma Patients with Worse Physical Function but Better Peer Relationships and Depressive Symptoms than U.S. Pediatric Population as Measured by PROMIS®

**DOI:** 10.7759/cureus.7040

**Published:** 2020-02-19

**Authors:** Anna R Cooper, Benjamin Wilke, Mark Scarborough, C. Parker Gibbs, Andre Spiguel

**Affiliations:** 1 Orthopaedic Surgery & Rehabilitation, Loyola University Medical Center, Maywood, USA; 2 Orthopaedics, Mayo Clinic, Jacksonville, USA; 3 Orthopaedic Surgery & Rehabilitation, University of Florida, Gainesville, USA; 4 Orthopaedics / Orthopaedic Surgery / Orthopaedic Oncology, University of Florida, Gainesville, USA

**Keywords:** bone and soft-tissue sarcoma, pediatric cancer, patient-reported outcomes, promis, limb-salvage surgery

## Abstract

Background

Pediatric patients with sarcomas are at risk of poor quality of life outcomes. The National Institutes of Health (NIH)-funded Patient-reported Outcomes Measurement Information System (PROMIS®) improves our ability to capture patient-reported outcomes. Do physical function, social, and mental health PROMIS outcomes for pediatric patients with non-metastatic malignant sarcomas differ from the U.S. pediatric population?

Methods

Six pediatric PROMIS short forms were collected for patient visits to orthopedic oncology at a tertiary referral center from September 1, 2016, to March 31, 2017. Mean T-scores differed from the reference population by a one-sample t-test.

Results

Of the 30 eligible patients, five had soft-tissue sarcomas and 25 (83%) had bone sarcomas. The mean age of the cohort was 13 years (5-17). The study cohort had a mean physical function T-score of 39.8 (SD 9.8), which was worse than the reference population. In contrast, the mean peer relationship T-score of 54.3 (SD 8.8) and mean depression T-score of 42.0 (SD 9.1) were better than the reference population.

Conclusions

Pediatric patients with non-metastatic sarcomas had a worse physical function but a better peer relationship and depression scores than the U.S. PROMIS reference population. Ceiling and flooring effects were reported. The level of evidence was III.

## Introduction

Pediatric patients with sarcomas are at risk of poor quality of life outcomes [1-2]. The management of their sarcomas often requires significant surgical interventions that alter mobility, physical function, and body image [3]. Patients are occasionally faced with complex decisions regarding limb-salvage resection or amputation and those treated with intensive chemotherapy are at risk of life-long effects [4-6].

The National Institutes of Health (NIH)-funded Patient-reported Outcomes Measurement Information System (PROMIS®) improves our ability to capture patient-reported outcomes in a standardized fashion [7-10]. The PROMIS pediatric measures were developed to capture the quality of life outcomes across a wide variety of health and disease states [11-15]. The pediatric PROMIS instruments have been tested in pediatric cancer populations and detected differences between survivors of cancer and patients currently actively treated. Survivors of childhood cancer reported better mobility, upper extremity functioning, and peer relationships than patients on active cancer treatment. Moreover, patients currently in treatment reported worse scores for depressive symptoms, anxiety, pain interference, and fatigue than survivors [16].

To the best of our knowledge, there are no prior reports of PROMIS pediatric outcomes for patients with non-metastatic sarcomas. Our study examined the following question: do physical function, social, and mental health PROMIS outcomes for pediatric patients with non-metastatic malignant sarcomas differ from the U.S. general pediatric population?

This article was presented at a conference: http://www.msts.org/view/download.php/education/pdfs/msts-2019-abstract-book.

## Materials and methods

Children and adolescents between the ages of five and 17 years were considered eligible for this study if they had a diagnosis of non-metastatic sarcoma, were literate in English, and able to complete electronic questionnaires. Patients included those with new diagnoses and those currently in treatment or surveillance. Exclusion criteria included non-oncologic and benign diagnoses, or metastatic disease, language barriers, and the inability to complete self-administered, computer-based questionnaires. Metastatic disease was defined as enlarging or at least 1-cm pulmonary nodules on chest CT, another osseous site of disease on whole-body bone scan, or lymph node disease as detected by positron emission tomography/computed tomography (PET/CT) and verified by histology. PROMIS questionnaires were routinely collected for all pediatric patient visits to the orthopedic oncology clinic at a tertiary referral center. Six static Pediatric PROMIS Short Forms were electronically administered on computers in the private clinic room. These questionnaires were administered as a standard of care for the practice and parents/guardians were permitted to remain present with the children but were informed not to influence the child’s responses. We excluded patients who opted for parental/guardian proxy short-form measures for this analysis. We retrospectively collected six months of data from September 1, 2016, to March 31, 2017. IRB approval (IRB201602495) was granted for this study. There was no funding source for this study. The patient's demographic and treatment data, as well as the patient-reported outcomes, were stored in a password-protected de-identified Excel (Microsoft Corporation, Redmond, Washington) file by the primary author. The dataset supporting the findings of this study are available by request to the corresponding author. Of the 164 pediatric patients who completed the questionnaires, 30 were eligible for this analysis with non-metastatic sarcoma diagnoses.

Patient-reported outcome measures

The PROMIS instruments have been studied in both general and clinical U.S. pediatric populations [11,14-20]. The short forms are targeted constructs of the complete item banks. All short forms use five-point Likert-style response categories to capture intensity, frequency, or duration. The instruments ask the patient to base responses on the seven-day recall to limit recollection bias. The instruments are publicly available and include scores manuals (http://www.healthmeasures.net/index.php?option=com_instruments&task=Search.pagination&Itemid=992) [21]. Derived scores were transformed to T-scores as referenced to the 2000 U.S. census on age, sex, and race/ethnicity [7-9].

PROMIS Pediatric Short Form v1.0 Physical Function Mobility 8a measures “self-reported capability rather than actual performance of physical activities” [21]. T-scores range from 15.2 to 58.2 and a higher score is interpreted as more of this measure, which is a positive outcome.

PROMIS Pediatric Short Form v2.0 Peer Relationships 8a assesses “quality of relationships with friends and other acquaintances.” Example questions include: “I felt accepted by other kids my age; Other kids wanted to talk with me” [21]. T-scores range from 17.7 to 64.4. A higher score represents more of this measure and is a positive outcome.

PROMIS Pediatric Short Form v2.0 Pain Interference 8a measures “consequences of pain on relevant aspects of one’s life.” This includes the extent to which pain hinders engagement with social, cognitive, emotional, physical, and reactional activities” [21]. T-scores range from 34 to 78 and a higher score represents more inhibition of activities due to pain, which is a negative outcome.

PROMIS Pediatric Short Form v2.0 Fatigue 10a assesses “a range of… symptoms, from mild subjective feelings of tiredness to an overwhelming, debilitating, and sustained sense of exhaustion that likely decreases one’s ability to execute daily activities and function normally in family or social roles” [21]. T-scores range from 31.1 to 82.8. A higher score indicates more of this measure and is a negative outcome.

PROMIS Pediatric Short Form v2.0 Depressive Symptoms 8a measures “negative mood (sadness, guilt), views of self (self-criticism, worthlessness), and social cognition (loneliness, interpersonal alienation), as well as decreased positive affect and engagement” [21]. T-scores range from 35.2 to 81.9. A higher score indicates more such symptoms and a negative outcome.

PROMIS Pediatric Short Form v2.0 Anxiety 8a measures “fear (fearfulness, panic), anxious misery (worry, dread), hyperarousal (tension, nervousness, restlessness), and somatic symptoms related to arousal (racing heart, dizziness)” [21]. Example questions are: “I felt like something awful might happen; I worried when I was at home. T-scores range from 32.2 to 82.8; a higher score indicating more of this measure is a negative outcome.

Statistical analysis

We reported descriptive statistics using chi-square for categorical variables and student’s t-test or analysis of variance (ANOVA) for continuous variables. We further assessed whether mean T-scores differed from the U.S. PROMIS pediatric reference population by the one-sample t-test. A post-hoc ANOVA was performed to examine the impact of surgical status on PROMIS T-scores by examining patients who completed the surveys preoperatively (n=7), those who did not have surgery as part of their management (n=3), and those who underwent surgery (n=20). All statistical analyses were performed with SPSS version 24.0 (SPSS Inc., Chicago, IL, USA). We considered p<0.05 significant.

## Results

Study patients

**Figure 1:**
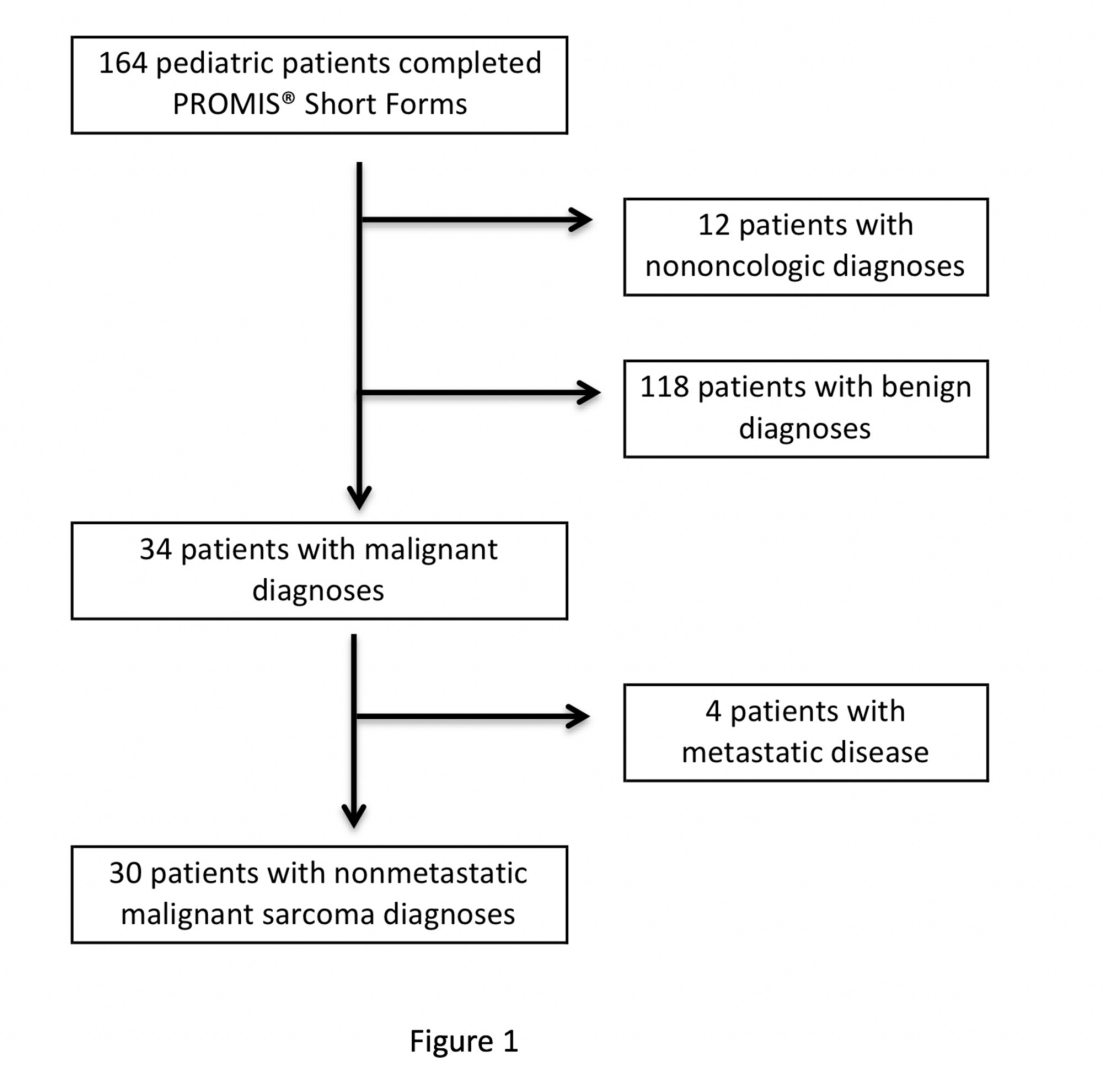
This flow chart depicts the patient selection process of non-metastatic bone and soft-tissue sarcoma pediatric patients. PROMIS: Patient-reported Outcomes Measurement Information System

A total of 30 patients were eligible for this study and the average age was 12.97 years (SD 2.77). The majority (n=26, 87%) were between 10 and 17 years of age. Fifty-three percent (n=16) were female. Primary bone sarcomas accounted for 83% (n=25) of the study population. The most common diagnosis was osteosarcoma (n=17, 57%) followed by Ewing sarcoma (n=7, 23%). Five patients (17%) were diagnosed with soft-tissue sarcomas. Patient selection is graphically depicted in Figure 1.

Relative to surgical intervention, seven (23%) completed the questionnaires prior to surgery and 20 (67%) were postoperative patients. The mean duration from surgery to the time of the questionnaire was 1.64 years (SD 1.61). Three patients experienced an incomplete resection prior to presenting to the practice. The majority of patients were treated with a limb salvage resection (n=24, 80%) and adjuvant therapy, e.g. chemotherapy or radiation therapy (n=26, 87%). Three patients experienced a major complication, e.g., requiring a return to the operating room (Table 1).

**Table 1:** The descriptive characteristics of the pediatric sarcoma patient cohort, including diagnostic and treatment variables, show the most common diagnosis was osteosarcoma. The majority of patients were treated with limb-salvage surgery and adjuvant therapy.

Characteristic		Distribution n (%)
Age, mean +/- SD years		12.97 (2.77)
Female Sex		16 (53)
Bone Sarcoma		
	Osteosarcoma	17 (57)
	Ewing Sarcoma	7 (23)
	Chondrosarcoma	1 (3)
Soft-Tissue Sarcoma		5 (17)
Preoperative Survey		7 (23)
Nonsurgical Management Only		3 (10)
Prior Incomplete Resection		3 (10)
Limb-Salvage Resection		24 (80)
Adjuvant (Chemotherapy and/or Radiation)		26 (87)
Postoperative Complication		3 (10)
Follow-up in Years if Surgical Candidate (N=20)		1.64 (1.61)

Outcome results

Descriptive statistics for the T-scores for the six PROMIS pediatric short-form outcomes are detailed in Table 2. The mean T-score for physical function mobility of 39.53 (SD 9.78) indicates less of the measure and a negative outcome. The subject responses were similar, as the interquartile range was 13 of a possible 43.3. There were three subjects whose responses were the maximum possible score, which is termed a ceiling effect. One patient was a 13-year-old who was 16 months from the resection of a proximal fibula chondrosarcoma. The second patient was a 13-year-old with a proximal humerus Ewing sarcoma who completed the questionnaire preoperatively. The third patient was a 15-year-old who was four months from the resection of a forearm synovial sarcoma.

**Table 2:** The mean, median, and quartile T-scores for six PROMIS outcome measures are shown for the cohort of non-metastatic sarcoma pediatric patients. The number and percentages of responses of the lowest (floor) and highest (ceiling) scores are included. PROMIS: Patient-reported Outcomes Measurement Information System

PROMIS Short Form Outcome Measure	Score Range	n	Mean	SD	25th Quartile	Median	75th Quartile	Floor	Ceiling
									n (%)	n (%)
Physical Function Mobility v1.0 (8a)	15.2-58.5	30	39.53	9.78	33	37.5	46	0 (0)	3 (10)
Peer Relationships v2.0 (8a)	17.7-64.4	30	54.27	8.82	47	54	65	0 (0)	8 (27)
Anxiety v2.0 (8a)	32.2-82.8	30	46.27	12.70	32	44	57.75	8 (27)	0 (0)
Depressive Symptoms v2.0 (8a)	35.2-81.9	30	42.03	9.07	35	40	44	13 (43)	0 (0)
Fatigue v2.0 (10a)	31.1-82.8	30	48.53	11.67	40	49.5	57.75	5 (17)	0 (0)
Pain Interference v2.0 (8a)	34-78	30	52.53	13.34	37.75	55.5	60.5	7 (23)	1 (3)

In contrast, the mean T-score for peer relationships was 54.27 (SD 8.82), indicating a positive outcome. There was a larger ceiling effect as eight (27%) patients scored the highest possible score on this questionnaire.

For the negatively worded outcomes of anxiety, depressive symptoms, fatigue, and pain interference, the study cohort had low T-scores for depressive symptoms. The mean T-score for depressive symptoms was 42.03 (SD 9.07), which was a positive outcome. Interestingly, this measure had the highest flooring rate with 13 patients (43%) scoring the lowest possible score. While the mean T-score for anxiety was not as low as for depressive symptoms, this measure also had flooring effects with eight patients (27%) scoring the lowest possible score for anxiety. Pain interference showed the widest spread of patient responses with an interquartile range of 22.75 (of a maximum 44) and the mean T-score was 52.53 (SD 13.34). There were seven patients (23%) who reported the lowest possible score and one patient who reported the highest possible score on this measure.

There were no significant differences in PROMIS T-scores based on surgical status. Patients who were surveyed preoperatively had a mean physical function T-score of 36.4 (SD 14.1), while those treated without surgery had a T-score of 40.7 (SD 16.3), and those who were treated with surgery had a T-score of 40.6 (SD 7.1); these did not differ from the overall mean of 39.5 (SD 9.8). The only measure that trended toward a difference between surgical status groups was pain interference (p=0.072). Unsurprisingly, this measure was higher in preoperative patients (mean 59.0, SD 10.6). It was even higher in those managed without surgery (mean 63.0) but with a wide standard deviation (13.8). Patients surveyed postoperatively had a mean T-score of 48.7 (SD 12.9). None of these statistically differed from the overall mean T-score of 52.5 (13.3).

Comparison to the U.S. pediatric reference population

Three measures were significantly different from the U.S. pediatric reference group: physical function mobility (p<0.0001), depressive symptoms (p<0.0001), and peer relationships (p=0.013, Table 3).

**Table 3:** The cohort mean T-scores for six PROMIS short-form measures were compared by the one-sample t-test to the U.S. general pediatric reference population. PROMIS: Patient-reported Outcomes Measurement Information System

PROMIS Outcomes	Mean	Standard Error of the Mean	t	Mean Difference	p (2-tailed)
Physical Function Mobility	39.53	1.78	-5.86	-10.47	0.000
Peer Relationships	54.27	1.61	2.65	4.27	0.013
Anxiety	46.27	2.32	-1.61	-3.73	0.118
Depressive Symptoms	42.03	1.66	-4.81	-7.97	0.000
Fatigue	48.53	2.13	-0.69	-1.47	0.497
Pain Interference	52.53	2.44	1.04	2.53	0.307

**Figure 2:**
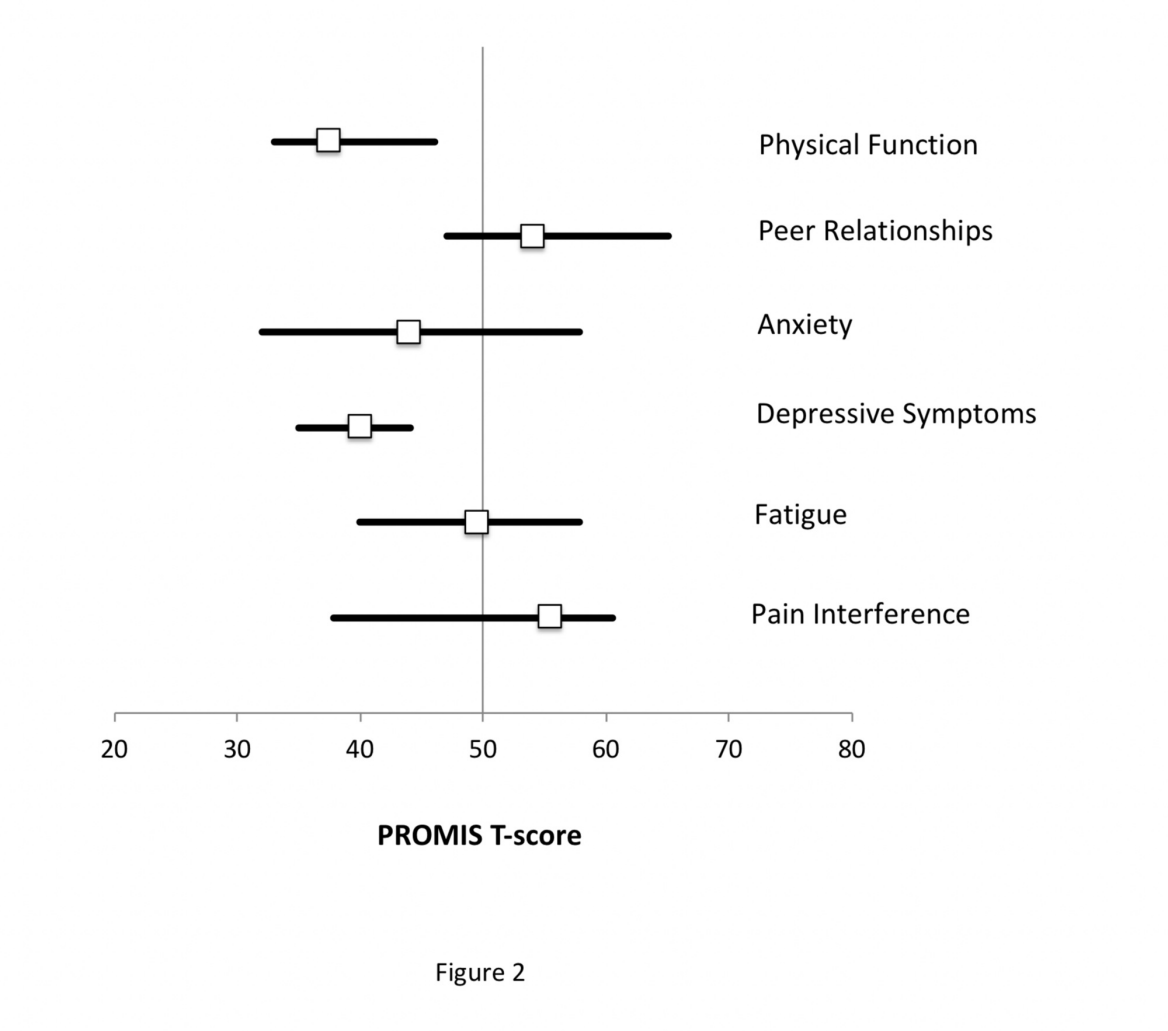
Median T-scores and interquartile ranges for each of the six PROMIS measures are shown for the study cohort relative to the U.S. population reference of 50. Physical function was significantly lower (indicating worse function) than the reference population and had a small interquartile range. Depressive symptoms were also significantly lower (indicating better symptoms) than the reference population and also had a small interquartile range. PROMIS: Patient-reported Outcomes Measurement Information System

These three measures displayed the least variability about the means as reflected by smaller standard errors of the mean. The box plot in Figure 2 graphically depicts the results by showing the median and quartile values for each PROMIS short-form measure.

## Discussion

In this study, pediatric patients with malignant bone and soft-tissue non-metastatic sarcomas indicated worse patient-reported outcomes related to physical function, but better outcomes related to peer relationships and depressive symptoms as compared to the U.S. pediatric reference population. This conclusion corroborates earlier quality of life studies of this rare patient population reporting diminished physical function outcomes [1,4-6,22].

To the best of our knowledge, this study is the first to examine PROMIS pediatric measures in sarcoma patients. In a meta-analysis, Stokke et al. examine the quality of life studies in pediatric, adolescent, and young adult patients with bone sarcoma. Of 452 identified manuscripts, 22 studies were examined for qualitative or quantitative analyses. The authors found heterogeneity of the study populations, quality of life instruments, and results such that their conclusions were limited. The authors call for the standardization of quality of life instruments in future studies [1]. As PROMIS is poised to potentially become the standardized quality of life instrument for patient-reported outcomes, it is important to examine these measures in this rare, specialized sarcoma population.

The current study provides a comparison to the U.S. reference group; in contrast, many earlier reports compare patients with extremity sarcomas treated with limb-salvage surgery to those treated with amputation [23-25]. For example, Ottaviani et al. examine survival, educational status, marital status, employment, and health insurance in patients with osteosarcoma diagnosed more than 20 years prior and compared those treated with limb-salvage procedures to those who underwent amputation. The cohort had a higher educational level and net income than the U.S. general population. The authors concluded patients adjusted well despite physical limitations in both treatment groups but do not offer a comparison of physical function to the U.S. general population [5].

In the current study, we found pediatric sarcoma patients had similar or better social health and mental health outcomes as compared with the U.S. pediatric reference population. Mental health outcomes in sarcoma patients yield a mixed picture in the literature [2-3,23-27]. These measures are paramount as reflected by a study by Siracuse et al. that found higher rates of suicide among adult patients with bone and soft-tissue sarcomas than the U.S. general population. As this study used Surveillance, Epidemiology, and End Results (SEER) data, granular level details regarding diagnosis and staging were not available [28]. One potential explanation for the discrepancy of our results is the positive effect of active cancer treatment. Our cohort was largely in the stages of active or recently completed therapy, times when patients feel most optimistic regarding prognosis and life expectancy; it will be important to consider temporal effects in future studies [26,29-30].

While the current study demonstrates the feasibility of implementing PROMIS short-form measures in an orthopedic pediatric population, there are several limitations. First, it is a small retrospective study. Additional subject numbers may alter the findings. In this retrospective analysis of prospectively collected data, selection bias may have occurred, as these data were drawn from a single tertiary institution. Therefore, our results may not be generalizable based on our population demographics and referral patterns.

Secondly, the cohort is a heterogenic group of rare diagnoses; however, the majority of the cohort consisted of malignant non-metastatic bone sarcomas (83%) and was managed with limb salvage resection (80%) and adjuvant chemotherapy and/or radiation (87%). Thus, it seemed appropriate to examine the outcomes as a composite group rather than by individual diagnoses. There was variability in tumor histology, location, and treatment that may impact the quality of life outcomes. This study was not sufficiently powered to examine these effects.

Third, the study does not include longitudinal data, and, therefore, we are unable to draw temporal conclusions. Each subject was surveyed once to maintain the independence of the responses but this design limits analyses examining a change over time or after a particular intervention. Our results did not show a difference between preoperative patients, postoperative patients, and those managed without surgery; however, this study was not powered to detect such differences. A future study of interest will be surveying patients preoperatively and then postoperatively as they recover from surgery and adjuvant treatments.

Finally, the performance of the PROMIS instruments in the study showed variability in responses, resulting in large standard errors and interquartile ranges for certain measures. In several measures, subjects responded with the lowest or highest possible score. When large proportions of the data are at the extremes of the outcome, this suggests the instrument may not be capturing the outcome sufficiently to have a reasonable variability in responses. Thissen et al. examined the minimally important difference (MID) in pediatric PROMIS measures for depression, pain interference, fatigue, and mobility/physical function and found the MID was approximately two points on the T-score [29]. This interpretation suggests the cohort had meaningful differences to the reference population on all measures except fatigue and pain interference. The interpretation of the PROMIS data will improve as our experience increases.

## Conclusions

Consistent with earlier outcome studies, this study of pediatric sarcoma patients showed decreased physical function measures but a positive psychosocial adjustment in peer relationships and depressive symptoms. In these measures, this study showed outcomes superior to the general reference population. This is consistent with studies suggesting the experience of surviving cancer improves coping skills and resilience.
